# When it just won’t go away: oral artemisinin monotherapy in Nigeria, threatening lives, threatening progress

**DOI:** 10.1186/s12936-017-2102-7

**Published:** 2017-12-15

**Authors:** ACTwatch Group, Louis Akulayi, Angela Alum, Andrew Andrada, Julie Archer, Ekundayo D. Arogundade, Erick Auko, Abdul R. Badru, Katie Bates, Paul Bouanchaud, Meghan Bruce, Katia Bruxvoort, Peter Buyungo, Angela Camilleri, Emily D. Carter, Steven Chapman, Nikki Charman, Desmond Chavasse, Robyn Cyr, Kevin Duff, Gylsain Guedegbe, Keith Esch, Illah Evance, Anna Fulton, Hellen Gataaka, Tarryn Haslam, Emily Harris, Christine Hong, Catharine Hurley, Whitney Isenhower, Enid Kaabunga, Baraka D. Kaaya, Esther Kabui, Beth Kangwana, Lason Kapata, Henry Kaula, Gloria Kigo, Irene Kyomuhangi, Aliza Lailari, Sandra LeFevre, Megan Littrell, Greta Martin, Daniel Michael, Erik Monroe, Godefroid Mpanya, Felton Mpasela, Felix Mulama, Anne Musuva, Julius Ngigi, Edward Ngoma, Marjorie Norman, Bernard Nyauchi, Kathryn A. O’Connell, Carolyne Ochieng, Edna Ogada, Linda Ongwenyi, Ricki Orford, Saysana Phanalasy, Stephen Poyer, Justin Rahariniaina, Jacky Raharinjatovo, Lanto Razafindralambo, Solofo Razakamiadana, Christina Riley, John Rodgers, Andria Rusk, Tanya Shewchuk, Simon Sensalire, Julianna Smith, Phok Sochea, Tsione Solomon, Raymond Sudoi, Martine Esther Tassiba, Katherine Thanel, Rachel Thompson, Mitsuru Toda, Chinazo Ujuju, Marie-Alix Valensi, Vamsi Vasireddy, Cynthia B. Whitman, Cyprien Zinsou, Chinazo Ujuju, Jennifer Anyanti, Paul N. Newton, Godwin Ntadom

**Affiliations:** 1 Population Services International, 1120 19th St NW Suit 600, Washington, DC 20036 USA; 2 Society for Family Health, No 8 Port Harcourt Crescent, Area 11, Garki, Abuja Nigeria; 3 London School of Hygiene and Tropical Medicine, 15-17 Tavistock Place, London, WCH 9SH UK; 4 Lao-Oxford-Mahosot Hospital-Wellcome Research Unit, Mahosot Hospital, Vientiane, Lao PDR & Centre for Tropical Medicine and Global Health, Nuffield Department of Clinical Medicine, University of Oxford, Oxford, UK; 5 National Malaria Elimination Programme, Federal Ministry of Health, Abuja, Nigeria

**Keywords:** Oral artemisinin monotherapy, Market share, Manufacturing, Anti-malarial products, Availability, Drug-resistance

## Abstract

**Background:**

Oral artemisinin monotherapy (AMT), an important contributor to multi-drug resistant malaria, has been banned in Nigeria. While oral AMT has scarcely been found for several years now in other malaria-endemic countries, availability has persisted in Nigeria’s private sector. In 2015, the ACTwatch project conducted a nationally representative outlet survey. Results from the outlet survey show the extent to which oral AMT prevails in Nigeria’s anti-malarial market, and provide key product information to guide strategies for removal.

**Results:**

Between August 10th and October 3rd, 2015 a total of 13,480 outlets were screened for availability of anti-malarials and/or malaria blood testing services. Among the 3624 anti-malarial outlets, 33,539 anti-malarial products were audited, of which 1740 were oral AMT products, primarily artesunate (n = 1731). Oral AMT was imported from three different countries (Vietnam, China and India), representing six different manufacturers and 11 different brands. Availability of oral AMT was highest among pharmacies (84.0%) and Patent Propriety Medicine Vendors (drug stores, PPMVs) (38.7%), and rarely found in the public sector (2.0%). Oral AMT consisted of 2.5% of the national anti-malarial market share. Of all oral AMT sold or distributed, 52.3% of the market share comprised of a Vietnamese product, Artesunat^®^, manufactured by Mekophar Chemical Pharmaceutical Joint Stock Company. A further 35.1% of the market share were products from China, produced by three different manufacturers and 12.5% were from India by one manufacturer, Medrel Pharmaceuticals. Most of the oral AMT was distributed by PPMVs accounting for 82.2% of the oral AMT market share. The median price for a package of artesunate ($1.78) was slightly more expensive than the price of quality-assured (QA) artemether lumefantrine (AL) for an adult ($1.52). The median price for a package of artesunate suspension ($2.54) was three times more expensive than the price of a package of QA AL for a child ($0.76).

**Conclusion:**

Oral AMT is commonly available in Nigeria’s private sector. Cessation of oral AMT registration and enforcement of the oral AMT ban for removal from the private sector are needed in Nigeria. Strategies to effectively halt production and export are needed in Vietnam, China and India.

**Electronic supplementary material:**

The online version of this article (10.1186/s12936-017-2102-7) contains supplementary material, which is available to authorized users.

## Background

In 2007, the World Health Organization (WHO) recommended that oral artemisinin monotherapy (AMT) should no longer be manufactured, produced, or distributed, given that its use leads to multi-drug resistant malaria [1]. Oral AMT was banned because of its role in development of malaria parasite resistance to artemisinin and its derivatives [2]. Artemisinin-resistant parasites are particularly problematic because the only effective therapy for *Plasmodium falciparum* malaria is artemisinin-based combination therapy (ACT); no alternative medicines are currently available. The loss of ACT efficacy would be disastrous for malaria endemic countries and would severely impede global malaria control and elimination progress [1]. Results from ACTwatch outlet surveys have shown that within a few years of the WHO recommendation, oral AMT was no longer available to consumers in multiple malaria endemic countries [3, 4]. The exception is ongoing availability and distribution in Nigeria, as described further in this paper, as well as in Myanmar, where oral AMT continues to be available and distributed despite the country’s national policy which has banned the use and importation of this anti-malarial [5–7].

In line with recommendations by WHO to remove oral AMT from the market, Nigeria banned oral AMT in 2006 [8]. Despite the ban, available evidence suggests that the National Agency for Food and Drug Administration and Control (NAFDAC) continues to issue product registration for oral AMT products as evidenced by NAFDAC registration numbers on product packaging. Furthermore, oral AMT products are marked by recent consumer protections to safeguard against fake anti-malarial medicines. Mobile authentication services for registered anti-malarial products have been extended to oral AMT products. For example, artesunate tablets manufactured by Mekophar Chemical Pharmaceutical Joint Stock Company in Vietnam were found carrying a Sproxil code for verification by SMS messaging (Additional file 1).

Results from previous national ACTwatch outlet surveys show that while oral AMT availability has declined in Nigeria’s private sector from 2009, when nearly half of private sector outlets were stocking oral AMT, in 2013 oral AMT was still found on the market with one in four private sector outlets stocking this anti-malarial [9]. This decline may in part be attributed to the aforementioned national ban on oral AMT, but also private sector initiatives designed to increase access to first-line ACT through a private sector co-payment mechanism.

The continued presence of oral AMT on the market in Nigeria threatens malaria control progress in a country with one of the highest contributions to the global malaria burden [5]. Furthermore, use of oral AMT in Nigeria could fuel parasite resistance to artemisinins as has occurred elsewhere in the world [10, 11]. Information about any continued availability of oral AMT in Nigeria, including product information to guide strategy, is therefore urgently needed.

ACTwatch was launched in 2008 by Population Services International (PSI) in collaboration with the London School of Hygiene and Tropical Medicine with support from the Bill and Melinda Gates Foundation (BMGF), the Department for International Development (DFID) and UNITAID. The goal of the project was to generate timely, relevant and high quality evidence on anti-malarial markets for policy makers, donors and implementing organisations [12]. As of 2016, ACTwatch had gathered data from a total of 12 malaria endemic countries in sub-Saharan Africa and the Greater Mekong Sub-region [3, 13]. Outlet surveys were designed to monitor availability, price and market share in the context of national strategies to improve access to national first-line ACT medicines, including but not limited to the private sector co-payment mechanism.

This article provides timely, relevant information regarding the availability and distribution of oral AMT in Nigeria in 2015, including relevant and specific product information. This information will be critical for national and international strategies to halt the continued manufacturing, export/import, and distribution of oral AMT in Nigeria.

## Methods

### Sampling

A nationally representative antimalarial outlet survey was conducted in Nigeria between August 10th and October 3rd, 2015. All categories of outlets with the potential to stock anti-malarials in both the public and private sector were included in the study. In the public sector, this included public health facilities (federal or state public facilities including teaching hospitals and federal medical centres at the tertiary level, general hospitals at the secondary level, and primary health centres and clinics at the primary level), community health workers (CHW) (including community health extension workers and role model mothers) and non-government, not-for-profit health facilities (hospitals and clinics). Outlets sampled in the private sector included private for-profit health facilities (hospitals, centres and clinics), pharmacies (licensed by the Pharmacy Council of Nigeria), drug stores [known as patent proprietary medicine Vendors (PPMVs)], general retailers selling fast-moving consumer goods, and itinerant drug vendors (mobile vendors without a fixed service delivery point).

As lists of all potentially eligible outlets were not routinely available, a cluster sampling approach with an outlet census in sampled clusters was used to identify outlets for inclusion [14, 15]. This entails sampling a set of administrative units with a population of approximately 10,000–15,000 inhabitants. In Nigeria, the most appropriate administrative unit matching this desired population size was a locality. A representative sample of localities was selected from with probability proportional to size within each of the six research domains: South–West, South–South, South–East, North–West, North–East, and North–Central. A stratified sampling approach was taken to produce estimates for each of these six geo-political zones. The sampling frame excluded areas with security issues that could threaten the safety of data collection teams.

The study was powered to detect change over time in the availability of quality-assured (QA) ACT (QAACT) and malaria blood testing. QAACT was defined as ACTs that achieved accredited status from the WHO, European Medicines Authority (EMA) or the Global Fund [16]. A series of calculations identified minimum sample size requirements to detect an increase or decrease in two key indicators: (1) proportion of outlets with QAACT available, among outlets with antimalarial(s) in stock on the day of the survey; and (2) proportion of outlets with malaria blood testing (RDT or microscopy) available, among outlets with antimalarial(s) in stock on the day of the survey or within the past 3 months. Calculations examined the sample size required to detect a 20-percentage point change between 2013 and 2015 among all outlets, public health facilities, and PPMVs.

The average numbers of outlets by facility type in localities within each geo-political zone screened during the 2013 outlet survey were used to estimate the number of clusters required in 2015 to achieve the desired sample sizes. Considering sample size requirements to detect change over time and average numbers of outlets across each outlet type, the optimal minimum number of localities required to reach desired numbers of outlets was 296 localities: n = 45 North–Central, n = 36 North–East, n = 59 North–West, n = 25 South–East, n = 31 South–South, n = 100 South–West.

Within selected clusters, a census of all outlets with the potential to sell or distribute anti-malarials and/or provide malaria blood testing was completed. To implement the census, interviewers systematically searched for all the aforementioned outlet types. Maps were used to identify the cluster boundaries and key informants were also used to ensure an exhaustive search was completed in each area. As outlets were identified, interviewers approached providers and were administered a series of screening questions to determine eligibility. Outlets eligible for the survey met at least one of three criteria: (1) one or more anti-malarials were in stock on the day of the survey, (2) one or more anti-malarials were in stock in the 3 months preceding the survey, and/or (3) malaria blood testing (microscopy or RDT) was available.

### Measures

The outlet survey was conducted using a paper questionnaire. Prior to implementing the study, the questionnaire was translated into three languages: Ibo, Hausa and Yoruba. The questionnaire was then back-translated into English to check the quality of the translation, and any inconsistencies were corrected.

Outlets meeting eligibility criteria noted above were invited to participate in the survey. Following informed consent procedures, an audit of all available anti-malarial treatments and RDTs was conducted. Anti-malarial audit information included formulation, package size, brand name, active ingredients and strengths, manufacturer, country of manufacture, reported sale/distribution in the week preceding the survey, retail price, and wholesale price. Up to three visits were made to all outlets to complete the screening process, audit, and provider interview, as needed (e.g. where outlets were closed or providers were not available).

### Training and data collection

A 5-day classroom-based interviewer training was implemented, and was followed by a 2-day field exercise to give newly trained interviewers practice with the study methodology and questionnaire. Extra participants for the training were recruited to ensure a sufficient number of interviewers, and to allow for a selection of best performing candidates as well as to accommodate any drop outs. Tests administered throughout the duration of the training were used to identify top performing interviewers. These candidates were then selected for a further 3 days of supervisor and quality controller training. During the interviewer training period, candidates who performed poorly after the training were either given additional reinforcement training or dropped from the data collection team.

When the data collection team arrived at the selected cluster, data collection team supervisors met with officials to crosscheck their list of public and any registered private sector outlets with that of the government list. Data collection teams verified locality boundaries with local leaders. The aforementioned census procedures were implemented to identify outlets. Data collectors interviewed the provider at the outlet after obtaining an informed consent. At the end of each day, questionnaires were reviewed by supervisors and quality controllers to identify any discrepancies or challenges with the quality of completed questionnaires. Spot checks were also conducted by quality controllers on 10–20% of the screened outlets. Outlets were randomly selected for spot checks by the supervisor, who then provided the outlet locations to the quality controllers. All physical questionnaires were ordered by cluster and serialized. These were then sent to Abuja for double data entry.

### Data preparation and analysis

A Microsoft Access (Microsoft Corporation, Redmond, Washington, USA) database with built-in range checks was used to conduct double data entry of physical questionnaires. Daily supervisor and data collector monitoring sheets were collated in a Microsoft Excel (Microsoft Corporation, Redmond, Washington, USA) spreadsheet, which along with the physical questionnaires, were used to cross-check entered data.

Standard ACTwatch indicators were calculated in line with previous outlet surveys [12, 14, 15]. Anti-malarials were classified as ACT, non-artemisinin therapy, and oral or non-oral artemisinin monotherapy. ACT were further classified as QAACT or non-quality assured ACT QAACT by matching product information to lists of WHO prequalified anti-malarials and Global Fund anti-malarial procurement lists.

Availability of oral AMT was calculated using the total number of outlets stocking any anti-malarial as the denominator. Market share was defined as the relative distribution of anti-malarials to individual consumers in the week preceding the survey. In order to allow for meaningful market share comparisons between products, information about anti-malarial distribution was standardized to the adult equivalent treatment dose (AETD). AETD is the amount of active ingredient necessary to treat a 60 kg adult according to WHO treatment guidelines [17]. Volumes distributed were calculated by converting provider reports on the number of anti-malarials sold in the week prior to the survey into AETDs. Volumes were therefore the number of AETDs sold or distributed by a provider in the 7 days prior to the survey. All dosage forms were considered when measuring volumes to provide a complete assessment of anti-malarial market share. Market share was also calculated on a sub-set of the data to illustrate the distribution of oral AMT products by manufacturer.

Anti-malarial price was collected in Nigerian Naira and converted to United States (US) dollars based on official exchange rates for the data collection period. The interquartile range (IQR) was calculated to demonstrate price dispersion. Price was calculated in two different ways. First, median private sector price per AETD was calculated for oral AMT, QAACT, chloroquine, and sulfadoxine–pyrimethamine (SP). AETD price measures included only tablet formulations given differences in unit costs for tablet and non-tablet formulations, which exist for anti-malarials other than QAACT. Second, the median price of oral AMT and QAACT was also presented as the price of pre-packaged therapy of oral AMT (typically 12 tablets of 50 mg; in comparison 1 AETD for artesunate 50 mg tablets is about 20 tablets), pre-packaged oral AMT for a child (i.e. 160 mg/80 ml, suspension), pre-packaged therapy for a 60 kg adult (i.e. AL 20/120, package size of 24 tablets), and pre-packaged therapy for a 10 kg child (i.e. AL 20/120 package size of 6 tablets).

All data cleaning and analysis was completed using Stata 13.1 (©StataCorp, College Station, TX). All point estimates were weighted using survey settings and all standard errors calculated taking account of the clustered and stratified sampling strategy with the relevant suite of survey commands in STATA.

## Results

### Sample

A total of 13,480 outlets were screened for availability of anti-malarials and/or malaria blood testing services. Of screened outlets, 3624 were stocking anti-malarials or testing on the day of the survey or within the past 3 months, and all of these outlets were subsequently interviewed. A total of 33,539 anti-malarials were audited, of which 1742 were oral AMT products (5% of all anti-malarial products audited).

### Availability

Table 1 shows the availability of oral AMT among anti-malarial stocking outlets in the public and private sector. Availability of oral AMT was higher in the private sector than the public sector (37.3% versus 2.0%, respectively) and was most commonly available among pharmacies (84.0%) followed by PPMVs (38.7%). Oral AMT was commonly available in the private sector in both urban (39.0%) and rural areas (36.4%) (Table 1).

**Table 1:** Availability of oral AMT, among outlets stocking at least one antimalarial, by outlet type

	Public health facility	Total public sector	Private for-profit facility	Pharmacy	PPMV (drug store)	General retailer	Total private sector
	% (95% CI)	% (95% CI)	% (95% CI)	% (95% CI)	% (95% CI)	% (95% CI)	% (95% CI)
National	N = 108	N = 204	N = 221	N = 225	N = 2701	N = 96	N = 3266
	1.8 (0.7, 4.6)	2.0 (0.9, 4.7)	17.1 (5.9, 40.3)	84.0 (73.1, 91.1)	38.7 (27.5, 51.2)	18.7 (6.4, 43.6)	37.3 (27.1, 48.8)
Urban areas	N = 98	N = 106	N = 177	N = 209	N = 2153	N = 78	N = 2632
	10.1 (2.7, 31.5)	9.8 (2.9, 28.4)	15.6 (8.2, 27.7)	87.1 (78.2, 92.7)	37.2 (30.1, 44.9)	31.0 (15.1, 53.3)	39.0 (32.2, 46.3)
Rural areas	N = 90	N = 98	N = 44	N = 16	N = 548	N = 18	N = 634
	0.8 (0.3, 2.6)	1.1 (0.4, 2.9)	17.6 (4.6, 48.5)	69.2 (34.9, 90.4)	39.5 (23.6, 58.0)	14.9 (2.6, 52.8)	36.4 (21.6, 54.3)

### Product information

Table 2 summarizes product information for audited oral AMT products. Of the 1740 oral AMT products audited with complete product information, almost all were artesunate products (n = 1731), with the exception of a few dihydroartemisinin products. The most commonly audited oral AMT product was from Vietnam, branded as Artesunat^®^, manufactured by Mekophar Chemical Pharmaceutical Joint Stock Company and formulated as tablets (n = 675) or suspensions (n = 470). Most of the other oral AMT products were from China, and produced by four different manufacturers, with most manufacturing two or more different brands. Both MD Artesunate^®^ (n = 213) and Actitesunate^®^ (n = 18) were manufactured by Jiangsu Ruinian Qianjin Pharmaceuticals; Gricin^®^ (n = 103) and Cusnat Artesunate^®^ (n = 33) were manufactured by Greenfield Pharmaceutical (Jiangsu) Co. Limited; Lever Artesunate^®^ (n = 82), Adamsnate^®^ (n = 31) and Codisin^®^ (n = 9) were manufactured by Adams Pharmaceutical Co. Ltd. Oral AMT was also imported from India, with the most commonly audited product, Aretmed^®^ (n = 99) manufactured by Medrel Pharmaceuticals (India) Private Ltd. (Table 3).

**Table 2:** Product catalogue of oral AMT products audited in Nigeria, 2015

Country of manufacture	Manufacturer	Brand	Package size	Strength (mg)	N
Artesunate tablet					
China	Adams Pharmaceutical (Anhui) Company Ltd.	Adamsnate	12	50	31
		Lever artesunate	12	50	82
	Greenfield Pharmaceutical (Jiangsu) Company Ltd.	Cusnat artesunate	12	50	33
		Gricin	12	50	103
	Jiangsu Ruinian Qianjin Pharmaceutical	Actitesunate	12	50	18
		MD artesunate	6	100	213
	Jiangxi Xierkangtai Pharmaceutical Company	Askasunate	12	50	6
India	Halex Pharmaceutical Pvt.	Vatunate	6	100	1
	Medrel Pharmaceutical (India) Pvt. Ltd.	Aretmed	12	50	99
Vietnam	Mekophar Chemical Pharmaceutical Joint Stock Company	Artesunat	12	50	675
Artesunate suspension					
	Mekophar Chemical Pharmaceutical Joint Stock Company	Artesunat	80 ml	160/80 ml	470
Dihydroartemisinin tablet					
China	Adams Pharmaceutical (Anhui) Company Ltd.	Codisin	8	60	3
Dihydroartemisinin suspension					
China	Adams Pharmaceutical (Anhui) Company Ltd.	Codisin	80 ml	160/80 ml	6
Total oral AMT					1740

**Table 3:** Median private sector price for anti-malarials

	N	USD	IQR
Price per AETD^a^			
Oral AMT	1228	$2.84	$2.44–$3.25
QAACT	8765	$1.69	$1.27–$2.44
SP	5745	$0.51	$0.46–$0.51
Chloroquine	1020	$0.25	$0.12–$0.49
Price per package			
Artesunate tablets	1226	$1.78	$1.52–$2.03
Artesunate suspensions	458	$2.54	$2.28–$3.04
QA AL-adult pack	1747	$1.52	$1.01–$1.78
QA AL-child package	1557	$0.76	$0.51–$1.01
AL suspension	1990	$3.04	$2.44–$3.55

^a^Adult equivalent treatment dose

Artesunate tablet products had a strength of 50 mg (12 tablets/blister) or 100 mg (6 tablets/blister). Oral AMT suspensions contained 160 mg/80 ml. Available photos of the products can be found in Additional file 2.

### Market share

**Fig. 1:**
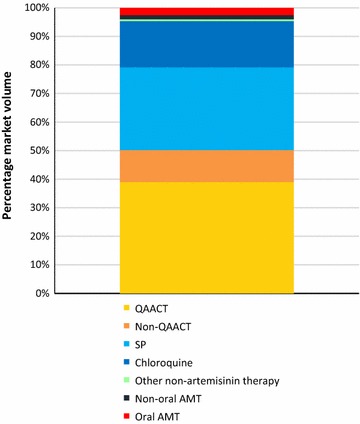
Anti-malarial market share

Figure 1 shows the national level anti-malarial market share for each type of anti-malarial distributed: ACT, non-artemisinin therapies, non-oral and oral AMT. About half of all anti-malarials distributed in Nigeria were ACT, including QAACT (39.0%) and non-QA ACT (11.2%). Other commonly distributed anti-malarials were non-artemisinins including SP (29.0%) and chloroquine (16.2%). Oral AMT accounted for 2.5% of the anti-malarial market nationally in 2015 (Fig. 1).

**Fig. 2:**
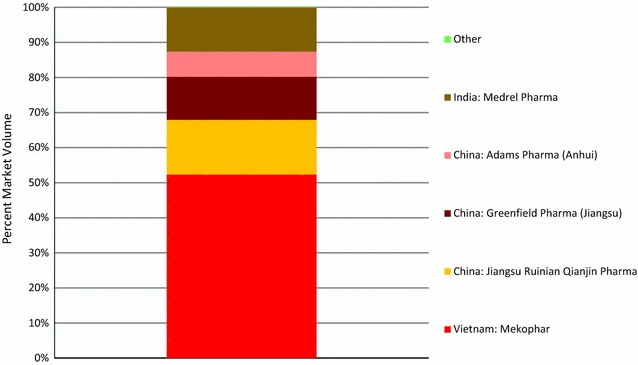
Oral AMT market share, by country of manufacturer and brand name

Figure 2 illustrates the market share of oral AMT by country of manufacturer and manufacturer. Of all oral AMT distributed, 52.3% of the market share was for a single brand manufactured in Vietnam by Mekophar. One in three oral AMT products distributed (35.1%) were manufactured in China by Jiangsu Ruinian Qianjin Pharmaceutical (12.3%), Greenfield Pharmaceutical (Jiangsu) (12.3%) or Adams Pharmaceutical (Anhui) (7.2%). In addition, 12.5% of the oral AMT products distributed were from India, manufactured by Medrel Pharmaceutical (Fig. 2).

**Fig. 3:**
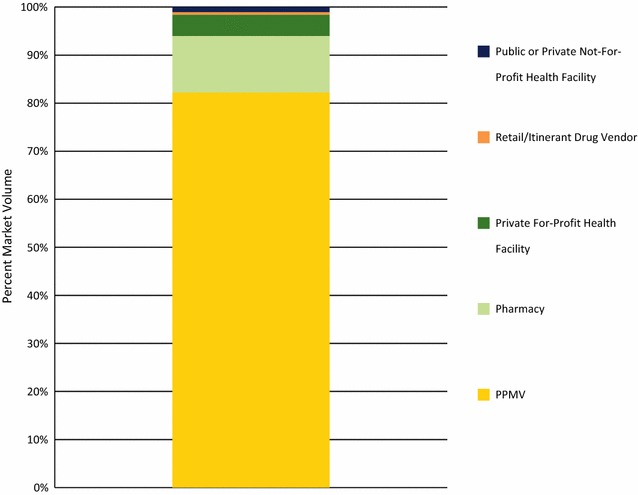
Public and private sector market share for oral AMT, by outlet type

Figure 3 illustrates the oral AMT market share for each outlet type. Most oral AMT was distributed by PPMVs accounting for 82.2% of oral AMT distributed, followed by pharmacies (11.8%) and private for-profit facilities (4.4%). Oral AMT was rarely distributed by the public sector (Fig. 3).

### Price

The median price of an AETD of oral AMT was $2.84 and up to 11 times more expensive than other anti-malarials: QAACT ($1.69), SP ($0.51) or chloroquine ($0.25) (Table 3).

The median price of pre-packaged artesunate tablets ($1.78) was only slightly more expensive than the price of pre-packaged QA AL for an adult ($1.52). The median price of pre-packaged artesunate suspension for a child ($2.54) was three times more expensive than the price of pre-package of QA AL for a child ($0.76) (Table 3).

## Discussion

Oral AMT is commonly available in Nigeria’s anti-malarial market, despite the country’s ban on this anti-malarial and calls from WHO to halt the importation and distribution of oral AMT. Findings on availability, distribution, and product information can inform the urgent action needed to remove products from the market.

### Oral AMT availability in the private sector

Oral AMT was commonly available in the private sector with over one in three outlets stocking at least one product on the day of the survey. The vast majority of pharmacies, and nearly 40% of PPMVs were stocking oral AMT. Strategies to rapidly remove oral AMT from the private sector should target these outlet types. Removing products from pharmacies may be less labor intensive than removal from PPMVs given that pharmacies are much fewer in number. PPMVs are ubiquitous, and furthermore, they were responsible for 76% of the total anti-malarial market share in 2015 [18], and distribute most of the oral AMT as evidenced in this study, making removal of oral AMT from their shelves critical.

Previous ACTwatch outlet surveys suggest that availability of oral AMT in Nigeria was in decline prior to 2015. In 2009, nearly half of all anti-malarial-stocking private sector outlets had oral AMT in stock (46%), and availability declined to 35% in 2011 and 25% in 2013 [4]. A continued decline in availability would be expected given the ban on oral AMT in place for several years prior to the 2015 survey [19]. However, it appears that oral AMT products continue to receive legal registration status granted by NAFDAC. NAFDAC registration numbers were found on the packages of common oral AMT products. While the National Malaria Elimination Programme (NMEP) within the Ministry of Health has banned oral AMT, halting product registration under NAFDAC has not yet been achieved. There is need to engage NAFDAC towards halting registration for these banned products. Several challenges have been documented with the registration process of medicines in Nigeria, including a lack of publically available lists of registered medicines, a lack of guidelines on the selection and retention of NAFDAC committee members, and an absence of conflict of interest forms for NAFDAC members [20]. It has been suggested that oral AMT is gradually being phased out by not registering any new oral AMT products and not renewing those with licenses that are due to expire [21]. Progress must be closely monitored to ensure that there is no new licensing of oral AMT products.

### Oral AMT distribution

Oral AMT accounted for a small percentage of all anti-malarial distribution; only 2.5% of all anti-malarials distributed were oral AMT products. This is an improvement over previous years when oral AMT market share was higher (2009, 8.1%; 2011, 4.1%). However, as of 2015, oral AMT market share had not improved over 2013 levels (2.0%). Continued oral AMT distribution must be addressed. Oral AMT is not the only set of anti-malarial medicines that is not indicated for malaria case management but continues to be widely available and distributed. It has been over a decade since the use of non-artemisinins including chloroquine and SP were phased out in favor of ACT for first-line treatment. Yet both chloroquine and SP retain a substantial market share, accounting together for nearly half of all anti-malarial distribution (45%). While SP is indicated for preventative therapy during pregnancy, its continued use for case management in Nigeria is evident in the high relative market share and product packaging promoting use to treat infection in people of all ages [3]. Furthermore, the use of these non-artemisinins, particularly chloroquine given concerns around efficacy, may pose greater threats to patient health and safety at least in the short term as compared to the longer term threats to drug efficacy posed by use of oral AMT. Strategies to remove oral AMT may be applied more broadly to ensure that other non-recommended anti-malarial medicines are removed from the market.

Chloroquine, SP and QAACT all had much higher relative market share as compared with oral AMT. This may be driven in part by price. Comparing the price of an adult equivalent treatment dose, oral AMT was 11 times more expensive than the least expensive treatment, chloroquine. A package of artesunate tablets was about $0.25 more expensive than an adult package of QA AL (first-line ACT treatment), thus the cost of oral AMT may in part explain lower levels of distribution. That said, it may also be the case that artesunate tablets are acquired and/or consumed in amounts much lower than a full course of treatment. This would make the overall cost of treatment with artesunate tablets perhaps comparable or cheaper than the cost of treatment with an ACT or popular non-artemisinin. The practice of dispensing and consuming artesunate tablets in amounts much lower than a full course has been documented in Myanmar, where oral AMT remains commonly available in the private sector [14, 15]. If this is indeed a common practice in Nigeria, the threats to patient health and safety and to the efficacy of artemisinin is high. Finally, if a consumer or patient was seeking treatment for a child, the results of the study also illustrate that artesunate formulated as a suspension was less expensive than a suspension of AL. In this instance, there may be a financial incentive for a consumer to choose the less expensive suspension, artesunate, over the first-line treatment for their child.

Finally, oral AMT products appeared to be marketed as powerful treatment that can cure drug-resistant malaria and severe malaria (see Additional file 3). It may be that these products are not competing directly with ACT and non-artemisinins for management of all types of malaria infection, but instead are directed towards those infections that are perceived to be drug resistant or severe.

### A diverse oral AMT product market

In contrast to other research which has shown that the distribution of oral AMT within a country is dominated by the presence of a single manufacturer and product on the market [7], this study found a diverse oral AMT market in Nigeria. Oral AMT products on the market in 2015 included 11 unique brands from six manufacturers and were imported from Vietnam, China and India. The Mekophar artesunate products from Vietnam dominated the market with about half of the oral AMT market share. However, four other manufacturers from China and India each held substantial proportions of market share. This finding suggests that the persistence of oral AMT products on the shelves in Nigeria is not the result of a single drug license or importation from one country. There may be a variety of existing agreements in place with manufacturers, importers and private buyers facilitating the presence of such a diverse set of products. Removing oral AMT products from the shelves will require addressing importation of multiple products from multiple manufacturers and three countries. Encouragingly, there are recent reports that a national task force has been established between the NMEP and NAFDAC to enforce the ban on oral AMTs [22]. However, to the authors’ knowledge, there is no further documentation publicly available that reports the remit of the task force or strategies that will be employed to enforce this ban.

Strategies to remove oral AMT must be prioritized and should be of imminent concern given the threats to malaria control and elimination posed by oral AMT consumption. Other countries that have successfully removed oral AMT from the market place have used a multi-faceted approach. For example, in Cambodia the National Malaria Control Programme took measures to improve enforcement of the oral AMT ban including private sector outlet inspections and confiscation of products performed by a new cadre of officials dedicated to enforcement of the ban [23]. This was complemented with a variety of activities to raise awareness of the ban targeting both providers and consumers, such as the use of posters to communicate about specific banned products and the dangers of using these products. Strategies in Cambodia were successful in removing oral AMT from the market [24]. The success may in part have been attributable to a concurrent private sector ACT subsidy program improving access to affordable first-line ACT treatment in the private sector [25]. Indeed, it may have been the private sector ACT subsidy programme implemented in Nigeria from 2010 to 2016 that led to initial reductions in market share for oral AMT [16]. Future strategies to remove oral AMT from the market in Nigeria must start with product registration as evidence suggests that oral AMT products are still granted legal registration by NAFDAC.

### Recommendations

Oral AMT products in Nigeria were labelled as originating from Vietnam, China and India. This is contrary to the World Health Assembly Resolution 60.18 from 2007 [26] and the authors call on these countries to comply with this in the interests of global public health. The last available WHO update on ‘Marketing of oral artemisinin-based monotherapy medicines—positions expressed by manufacturers’ is from December 2015 [27]. Only two of the seven manufacturers listed in Table 2 are named in this document. The authors call on the Global Malaria Programme to update this and for the new Director-General to lead discussions on how to end this practice—that is especially dangerous for malaria endemic Africa. If needed this issue should be brought before the World Health Assembly in 2018 to give countries continuing to manufacture oral AMT the opportunity to explain their manufacturing policy in relation to global malaria control.

At a national level, additional enforcement strategies should be implemented to ensure full removal or oral AMT from the market. For these to be most effective, there is need for coordination between the National Malaria Elimination Programme and NAFDAC to ensure that banned products are no longer legally registered. In addition, it is recommended that efforts focus on the removal of registered and unregistered products from the private sector, particularly by targeting PPMVs where oral AMT is most commonly available and distributed.

Finally, the results from the study in Nigeria show the need for further information about supply practices and supply chains for oral AMT products, as well as information about provider and consumer preferences and practices. In particular, understanding consumer and provider choice and perceptions around the efficacy of different types of anti-malarials may help to explain why oral AMT continues to be distributed and administered by providers.

## Limitations

The outlet survey includes an audit of all anti-malarials by asking the provider to show the interviewer all treatments that are in stock. Given that oral AMT has been banned in Nigeria, providers may have been reluctant to show oral AMT products to interviewers. Data from this study may, therefore, produce underestimates for the extent to which oral AMT is available and is contributing to market share. The outlet survey is a tool to provide estimates of availability, price and market share. While beyond the scope of this study, qualitative information on provider awareness of the ban and consumer preferences would have added valuable contextual information to assist with the interpretation of findings.

## Conclusion

Nigeria has one of the highest burdens of malaria in the world, accounting for an estimated 23% of cases globally and one-third of all deaths. As such, the availability and distribution of oral AMT is of grave concern and poses a serious threat to malaria control in Nigeria and the rest of the world. The evidence from this study illustrates that oral AMT is available and distributed by the private sector, namely though PPMVs, and comprises of 11 different products manufactured by three countries—India, Vietnam and China. Strategies to remove oral AMT from the market in Nigeria must start with product registration as evidence suggests that oral AMT products are still granted legal registration by NAFDAC. Strategies are required to remove registered and unregistered products from the private sector, particularly by targeting PPMVs where oral AMT is most commonly available and distributed. Finally, there is urgent need for action in Vietnam, China and India where oral AMT is still manufactured and exported.

## Additional files

**Additional file 1.** Photos of oral AMT products found in Nigeria’s 2015 ACTwatch outlet survey with NAFDAC registration numbers.

**Additional file 2.** Product information and photos of oral AMT products found in Nigeria’s 2015 ACTwatch outlet survey.

**Additional file 3.** Photos of oral AMT packaging and product inserts noting use for resistant malaria found in Nigeria’s 2015 ACTwatch outlet survey.

## Abbreviations

AETD: adult equivalent treatment dose
ACT: artemisinin-based combination therapy
AL: artemether-lumefantrine
BMGF: Bill and Melinda Gates Foundation
Co: company
DFID: Department for International Development
IPTp: intermittent treatment as prevention during pregnancy
IQR: interquartile range
LGA: local government areas
Ltd: limited
NAFDAC: National Agency for Food and Drug Administration and Control
NMEP: National Malaria Elimination Programme
NGO: non-government organization
SP: sulfadoxine–pyrimethamine
PPMV: patent proprietary medicine vendors
PSI: Population Services International
QA: quality-assured
WHO: World Health Organization
