# Supplementary material for: When it just won’t go away: oral artemisinin monotherapy in Nigeria, threatening lives, threatening progress
Source: Malar J. 2017 Dec 15;16:489. doi: 10.1186/s12936-017-2102-7 (PMC5732368; doi:10.1186/s12936-017-2102-7)

Additional file 1: Photos of oral AMT products found in Nigeria’s 2015 ACTwatch outlet survey with NAFDAC registration numbers

Artesunat made by Mekophar Chemical Pharmaceutical Joint-Stock Company, Vietnam

NAFDAC Registration Number: 04-3397.


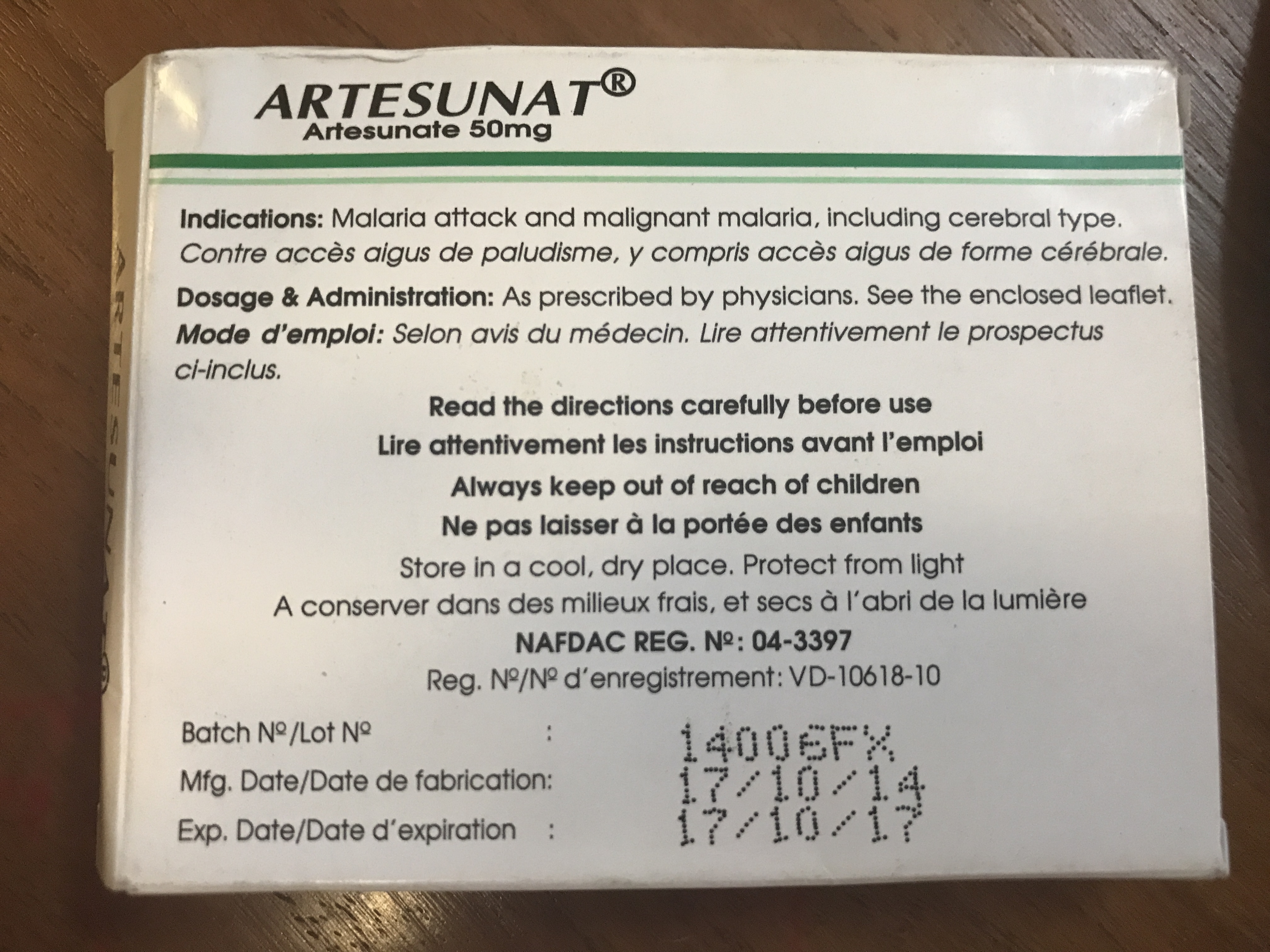


MD Artesunate made by Jiangsu Ruinian Qianjin Pharmaceutical Co. LTD, China

NAFDAC Registration Number: A4-9104


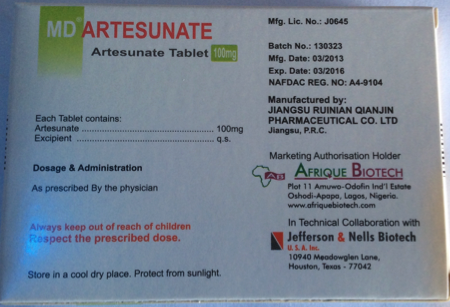


Artemed made by Medrel Pharmaceuticals (India) Pvt. Ltd., India

NAFDAC Registration Number: 04-4213


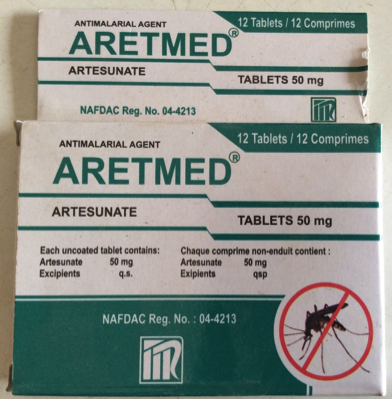


Artesunat made by Mekophar Chemical Pharmaceutical Joint-Stock Company, Vietnam

Product contains a Sproxil mobile authentication service code. An SMS message sent to the indicated number with the specified code yielded this authentication message from NAFDAC noting “genuine artesunate tablet.” June, 2015.


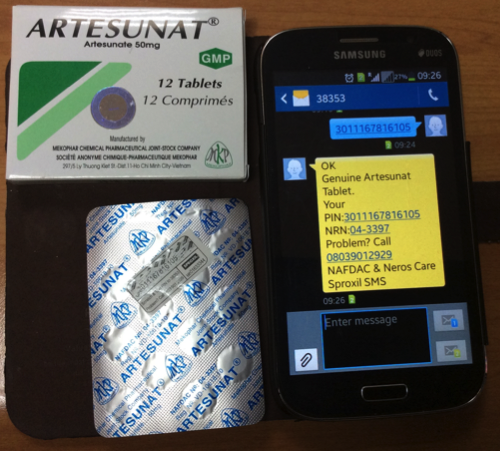

Supplement: Supplementary file 1 — Additional file 1. Photos of oral AMT products found in Nigeria’s 2015 ACTwatch outlet survey with NAFDAC registration numbers. [file 12936_2017_2102_MOESM1_ESM.docx]
