# Supplementary material for: When it just won’t go away: oral artemisinin monotherapy in Nigeria, threatening lives, threatening progress
Source: Malar J. 2017 Dec 15;16:489. doi: 10.1186/s12936-017-2102-7 (PMC5732368; doi:10.1186/s12936-017-2102-7)
Supplement: Supplementary file 2 — Additional file 2. Product information and photos of oral AMT products found in Nigeria’s 2015 ACTwatch outlet survey. [file 12936_2017_2102_MOESM2_ESM.docx]

Additional file 2: Product information and photos of oral AMT products found in Nigeria’s 2015 ACTwatch outlet survey

Country of Manufacture: China

| **Brand** | **Formulation** | **Active Ingredient : Strength** | **Manufacturer** | **Photo** |
| --- | --- | --- | --- | --- |
|  |  |  |  |  |
| Actitesunate | Tablet | Artesunate : 50mg | Jiangsu Ruinian Qianjin Pharmaceutical | No photo available |
|  |  |  |  |  |
| Adamsnate | Tablet | Artesunate : 50mg | Adams Pharmaceutical (Anhui) Co. Ltd. | 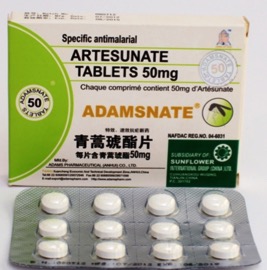 |
|  |  |  |  |  |
| Askasunate | Tablet | 50mg | Jiangxi Xierkangtai Pharmaceutical Co. Ltd. | 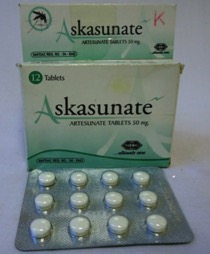 |
|  |  |  |  |  |
| Cusnat Artesunate | Tablet | Artesunate : 50mg | Greenfield Pharmaceutical (Jiangsu) Co. Ltd. | No photo available |
|  |  |  |  |  |
| Gricin | Tablet | Artesunate : 50mg | Greenfield Pharmaceutical (Jiangsu) Co. Ltd. | No photo available |

| **Brand** | **Formulation** | **Active Ingredient : Strength** | **Manufacturer** | **Photo** |
| --- | --- | --- | --- | --- |
| Lever Artesunate | Tablet | Artesunate : 50mg | Adams Pharmaceutical (Anhui) Co. Ltd. | 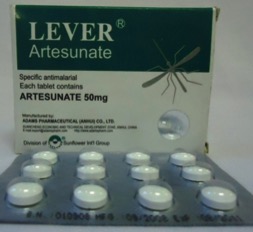 |
|  |  |  |  |  |
| MD-Artesunate | Tablet | 100mg | Jiangsu Ruinian Qianjin Pharmaceutical Co. Ltd. | 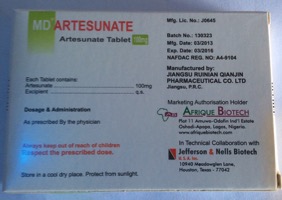 |
|  |  |  |  |  |
| Codisin | Tablet | Dihydro-artemisinin: 60mg | Adams Pharmaceutical (Anhui) Co. Ltd. | 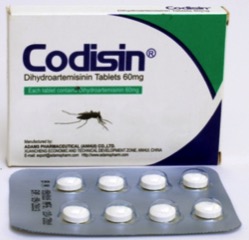 |
|  |  |  |  |  |

| **Brand** | **Formulation** | **Active Ingredient : Strength** | **Manufacturer** | **Photo** |
| --- | --- | --- | --- | --- |
| Codisin | Suspension | Dihydro-artemisinin: 160mg per 80ml | Adams Pharmaceutical (Anhui) Co. Ltd. | 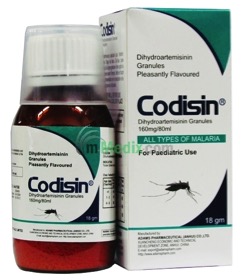 |

Country of Manufacture: India

| **Brand** | **Formulation** | **Active Ingredient : Strength** | **Manufacturer** | **Photo** |
| --- | --- | --- | --- | --- |
|  |  |  |  |  |
| Artemed | Tablet | Artesunate : 50mg | Medrel Pharmaceuticals (India) Pvt. Ltd. | 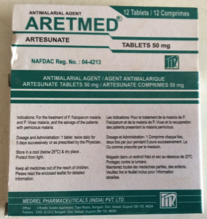 |
|  |  |  |  |  |
| Vatunate | Tablet | Artesunate : 100mg | Halex Pharma | No photo available |

Country of Manufacture: Vietnam

| **Brand** | **Formulation** | **Active Ingredient : Strength** | **Manufacturer** | **Photo** |
| --- | --- | --- | --- | --- |
|  |  |  |  |  |
| Artesunat | Tablet | Artesunate : 50mg | Mekophar Chemical Pharmaceutical Joint-Stock Company | 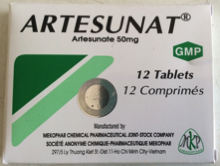 |
|  |  |  |  |  |
| Artesunat | Suspension | Artesunate : 160mg / 80ml | Mekophar Chemical Pharmaceutical Joint-Stock Company | 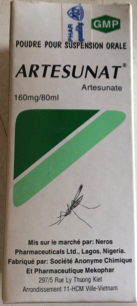 |
