# Supplementary material for: When it just won’t go away: oral artemisinin monotherapy in Nigeria, threatening lives, threatening progress
Source: Malar J. 2017 Dec 15;16:489. doi: 10.1186/s12936-017-2102-7 (PMC5732368; doi:10.1186/s12936-017-2102-7)

Additional file 3: Photos of oral AMT packaging and product inserts noting use for resistant malaria found in Nigeria’s 2015 ACTwatch outlet survey

Artesunat made by Mekophar Chemical Pharmaceutical Joint-Stock Company, Vietnam

Product insert noting indication “…specifically against parasites resistant to Chloroquine.” And “…control the acute attack and cerebral type of malaria.”


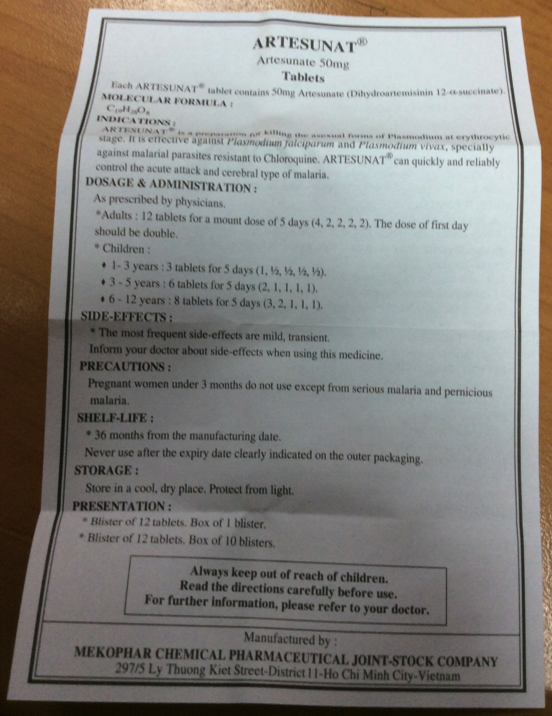


MD Artesunate made by Jiangsu Ruinian Qianjin Pharmaceutical Co. LTD, China

Package noting indication “…the treatment of malaria caused by all forms of plasmodium including multi-drug resistant strains of P. falciparum.”


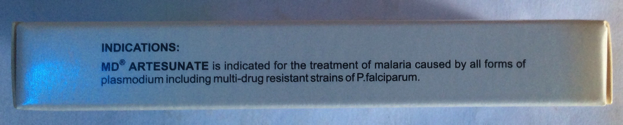

Supplement: Supplementary file 3 — Additional file 3. Photos of oral AMT packaging and product inserts noting use for resistant malaria found in Nigeria’s 2015 ACTwatch outlet survey. [file 12936_2017_2102_MOESM3_ESM.docx]
